# Devolution and public perceptions and experience of health services in Pakistan: linked cross sectional surveys in 2002 and 2004

**DOI:** 10.1186/1472-6963-11-S2-S4

**Published:** 2011-12-21

**Authors:** Umaira Ansari, Anne Cockcroft, Khalid Omer, Noor MD Ansari, Amir Khan, Ubaid Ullah Chaudhry, Neil Andersson

**Affiliations:** 1 CIET in Pakistan, PO Box 13018, Karachi 75350, Pakistan; 2 CIET Trust Botswana, PO Box 1240, Gaborone, Botswana; 3 Department of geography and urban regional planning, University of Peshawar, Peshawar, Pakistan; 4 Centro de Investigación de Enfermedades Tropicales, Universidad Autónoma de Guerrero, Acapulco, Mexico

## Abstract

**Background:**

The government of Pakistan introduced devolution in 2001. Responsibility for delivery of most health services passed from provincial to district governments. Two national surveys examined public opinions, use, and experience of health services in 2001 and 2004, to assess the impact of devolution on these services from the point of view of the public.

**Methods:**

A stratified random cluster sample drawn in 2001 and revisited in 2004 included households in all districts. Field teams administered a questionnaire covering views about available health services, use of government and private health services, and experience and satisfaction with the service. Focus groups in each community discussed reasons behind the findings, and district *nazims* (elected mayors) and administrators commented about implementation of devolution. Multivariate analysis, with an adjustment for clustering, examined changes over time, and associations with use and satisfaction with services in 2004.

**Results:**

Few of 57,321 households interviewed in 2002 were satisfied with available government health services (23%), with a similar satisfaction (27%) among 53,960 households in 2004. Less households used government health services in 2004 (24%) than in 2002 (29%); the decrease was significant in the most populous province. In 2004, households were more likely to use government services if they were satisfied with the services, poorer, or less educated. The majority of users of government health services were satisfied; the increase from 63% to 67% between 2002 and 2004 was significant in two provinces. Satisfaction in 2004 was higher among users of private services (87%) or private unqualified practitioners (78%). Users of government services who received all medicines from the facility or who were given an explanation of their condition were more likely to be satisfied. Focus groups explained that people avoid government health services particularly because of bad treatment from staff, and unavailable or poor quality medicines. District *nazims* and administrators cited problems with implementation of devolution, especially with transfer of funds.

**Conclusions:**

Under devolution, the public did not experience improved government health services, but devolution was not fully implemented as intended. An ongoing social audit process could provide a basis for local and national accountability of health services.

## Background

At the beginning of the 21^st^ century, even compared with its neighbours in South Asia, Pakistan had poor health indicators. Government primary care health facilities were under-used and most of the population relied on the private sector (including unqualified and traditional practitioners) for basic health care [1]. The local government plan promulgated in 2000 by the military government of President Pervez Musharaff [2] aimed to extend democracy at local levels, to increase accountability, and to improve delivery of public services including health care. New arrangements under devolution were intended to strengthen the role of district governments; new posts for elected majors (*nazims*) were created at district and sub-district levels. A key intention of devolution was to improve the lot of disadvantaged members of the society, such as women and the very poor [2]. Prior to devolution, the delivery of health services was the responsibility of the four provincial governments; under the local government plan, the provinces remained responsible for planning and monitoring of health services but delivery of most health services, including management of human resources for health, became a district function [3]. Tertiary hospital services remained under provincial control and some vertical programmes remained federal responsibilities, like the Lady Health Worker programme, and programmes for malaria, tuberculosis, and HIV/AIDS [3].

In order to assess the impact of the new local government system from the standpoint of ordinary Pakistani citizens, the National Reconstruction Bureau commissioned an independent social audit process to seek the views and experience of the public about local democracy and several key government services. We carried out two national surveys, in 2001-2002 at the beginning of devolution, and in 2004 three years into devolution. We produced local reports on the findings and these contributed to the debate within Pakistan about the impact of devolution [4,5]. In this paper we describe the social audit methods and the findings about health services, and discuss the implications of the findings in relation to devolution.

## Methods

The Human Research Review Committee of HOPE (Health Oriented Preventive Education), based in Karachi, Pakistan, reviewed and gave ethical approval for the surveys.

### Sample

For the 2001-2 survey a stratified random cluster sample included clusters in all the (then) 97 districts in the country, based on the population frame of the 1998 census. We randomly selected union councils (the smallest administrative unit) in each district, with the number of councils in each district set proportional to the census urban/rural balance in the district. In the more populous districts, mainly in Punjab province, we selected up to eight union councils. We then randomly selected one community (village or ward) within each selected union council. In each selected community, interviewers contacted all contiguous households up to 120, radiating from a randomly allocated starting point. There was no sub-sampling within each site selected: all households were included. We defined a household as people living together, sharing a kitchen and eating together. The 2004 survey used the same sample as in the 2001/2 survey, with the addition of sites in newly formed districts, to a total of 101 districts at the time of the survey.

### Instruments and data collection

The CIET team in Pakistan developed the instruments for the social audit in consultation with stakeholders in Pakistan, including the government agency responsible for the design and implementation of devolution. The *household questionnaire* for the 2001/2 survey, initially in English, was translated and back-translated it into the local languages of different parts of the country. We piloted the questionnaire in non-sample sites and made adjustments to improve interpretation and flow. A general section, administered to the household head or a senior household member, covered socio-economic status, demographics, and views about key public services. Further sections covered views and experience about several public services. The section on health services asked which service the household members usually used for treatment of health problems, about access to this service, and about self-reported knowledge of how to complain about the service (without asking what the method of complaining was). It further asked about the experience of the service on the last occasion when it was used by any family member, where possible getting this information directly from the family member concerned (or the carer in the case of a child): presence of a doctor; explanation about the condition; availability of medicines in the facility; payments for elements of the service; and satisfaction with the service received. The 2004 survey questionnaire asked the same questions about health services as in 2001/2.

A *community profile questionnaire*, administered to a key informant (typically a shop keeper) in the community, collected information about features relevant to the use and experience of health facilities, such as the distance from government and other health care facilities. *Key informant questionnaires* in 2004 sought information from district *nazims* (elected mayors) and district coordinating officers (DCOs – appointed civil service administrators) about the implementation of devolution in the district.

Findings from the initial analysis of the 2004 household survey were the basis for a *focus group guide* to feedback and discuss key findings with separate focus groups of men and women in each sample community.

Local field teams, comprising both male and female members, underwent a combination of classroom and practical instruction from Pakistani CIET personnel, who were also responsible for supervision of all fieldwork. Data collection for the household survey took place over about four months for each survey. Trained members of the teams returned to the districts and sample communities a few months later to conduct the interviews with the district *nazims* and DCOs (for the later survey only) and the focus group discussions. In each site they attempted to conduct two focus groups, male and female, with 8-10 participants in each group. The facilitator guided the discussion while the reporter took detailed notes.

### Analysis

Data entry operators in Karachi used Epi Info to enter data twice, with validation to minimise key stroke errors. Further cleaning looked for logical errors, out of range responses and duplications, checking back to the original data registers as necessary. Analysis relied on CIETmap open source software that combines epidemiological analysis with raster and vector mapping [6].

We calculated site weights to allow for non-proportional sampling across the provinces, in particular over-sampling of districts in sparsely-populated Balochistan, and used these to calculate weighted national and provincial figures for measured outcomes. A vulnerability index for economic status combined information about the type of roof construction, room occupancy and occupation of the main household breadwinner. A household counted as *vulnerable* if it had at least two of the following three factors: poor roof construction, overcrowding or poor occupation of the main breadwinner. If all three factors were present, the household counted as *very vulnerable*. The analysis of the experience and satisfaction of health service users focused on the last family member to use a health service within the last three months, in order to increase the chances of accurate recall of the service contact.

We examined associations between outcomes of interest (households' views and use of government health services, and users' experience and satisfaction with the services) and potentially associated variables, first in bivariate analysis and then in multivariate analysis using the Mantel Haenszel procedure [7], with an adjustment for clustering described by Gilles Lamothe, based on a variance estimator to weight the Mantel Haenszel odds ratio for cluster-correlated data [8,9]. Multivariate analysis began with saturated models including all variables potentially associated with the outcome, and stepped down to final models including only variables that remained significantly associated with the outcome. Initial models for household views and use of services included: sex and education of the respondent; education of the household head; vulnerability of the household; urban or rural location; and proximity of the nearest government health facility. Initial models for satisfaction of government service users included: vulnerability of the user's household; urban or rural location; payments made for medicines and to health workers; explanation of the condition; and availability of required medicines. Because there was important heterogeneity between provinces and interaction between province and a number of other variables in overall analyses, we undertook separate analyses for each province. To allow for the large sample size and multiple comparisons, we used the 99% confidence level for statistical significance. Associations are expressed as the adjusted Odds Ratio (ORa) and its cluster-adjusted 99% confidence interval (CIca).

A small group reviewed the focus group reports in the relevant local languages. They identified themes about why people avoided using government health services and about what could be done to encourage people to use these services, and extracted relevant quotes.

## Results

Table 1 shows some characteristics of the 57,321 households interviewed in 2001/2 and 53,960 interviewed in 2004. Over one half of the household respondents were women. Nearly all the household heads were male and about one half of them had some formal education. Nearly half the households were categorised as vulnerable (with at least two out of three vulnerability factors) while about one in eight were categorised as very vulnerable (with all three of the vulnerability factors). The characteristics of the sample households were similar in 2001/2 and 2004.

**Table 1:** The households included in the survey in 2001/2 and 2004

Characteristic	Weighted percentage (fraction) of households	
	2001/2 (n=57,321)	2004 (n=53,960)
Urban households	34.8 (15,552/57,321)	35.8 (15,133/53,960)
Household respondent female	54.1 (24,626/57,294)	54.3 (27,594/53,948)
Household head male	87.0 (47,775/57,311)	93.2 (51,138/53,953)
Household head with any formal education	50.8 (26,588/57,223)	53.2 (25,792/53,848)
Household vulnerable^1^	44.2 (29,876/56,251)	47.5 (28,048/52,784)
Household very vulnerable^2^	13.2 (8,984/56,251)	13.4 (7,701/52,784)

^1^ Vulnerable households had at least two of three vulnerability factors: poor roof construction, overcrowding, and poor occupation of the main breadwinner.

^2^ Very vulnerable households had all three vulnerability factors.

### Household opinions and use of government health services

Less than a quarter of households in 2002 (22.8% weighted, 10,429/46,396) were satisfied with government health services in their area, ranging from 17% in Balochistan (1,748/10,069) to 27% (2,672/10,688) in Khyber Pakhtunkwa (formerly North West Frontier Province). In 2004, slightly more households were satisfied with government health services (26.9% weighted, 13,117/53,381); the difference between 2002 and 2004 was not significant at the 1% level in any province.

Table 2 shows findings from the final models of multivariate analysis of variables associated with household satisfaction with the government health services in their area in 2004. Findings were broadly similar across the provinces. In all provinces, male respondents were less likely to say they were satisfied. In Balochistan and Khyber Pakhtunkhwa, poorer households (those categorised as vulnerable) were less likely to be satisfied. Respondents with any formal education were more likely to say they were satisfied with government health services, except in Punjab, and in Balochistan respondents from a household where the head had some formal education were also more likely to be satisfied. Households who reported they knew how to complain about their usual health service were more likely to be satisfied in Balochistan and Punjab. Proximity to a government health facility did not make any difference to household satisfaction, except in Punjab where those residing within 5 Km of a government health facility were more likely to be satisfied.

**Table 2:** Final models from multivariate analysis of variables associated with household satisfaction with government health services in 2004, in the four provinces

	ORa	99% CIca
**Explanatory variable/Province**		

**Sindh**		
Male respondent	0.71	0.56 – 0.91
Respondent with any formal education	1.39	1.03 – 1.89
**Balochistan**		
Male respondent	0.58	0.36 – 0.69
Respondent with any formal education	1.26	1.01 – 1.62
Household head with any formal education	1.67	1.17 – 1.66
Vulnerable household^1^	0.59	0.55 – 0.88
Know how to complain about the service	1.26	1.06 – 1.78
**Khyber Pakhtunkhwa**		
Male respondent	0.64	0.43 – 0.78
Respondent with any formal education	1.15	1.09 – 1.65
Vulnerable household^1^	0.73	0.65 – 0.94
**Punjab**		
Male respondent	0.85	0.74 – 0.98
Know how to complain about the service	1.21	1.05 – 1.43
Government health facility within 5 Km	2.18	1.27 – 3.66

OR= adjusted Odds Ratio;

CIca= cluster adjusted confidence interval around the ORa

^1^ Vulnerable households had at least two of three vulnerability factors: poor roof construction, overcrowding, and poor occupation of the main breadwinner.

The variables included in all initial models were: urban/rural location of household, sex of the respondent, education of the respondent, education of the household head, and vulnerability status of the household, knowledge of how to complain about health services, whether there was a government health facility within 5 Km.

Less than a third of households usually used a government health facility for medical attention in 2002, and this proportion was even lower in 2004 (Table 3). The commonest source of health care in both surveys was private qualified practitioners. About a quarter of households usually used unqualified medical practitioners in 2002 and this proportion was higher in 2004. There was considerable variation among the four provinces in use of government health facilities (Table 4). In both 2001/2 and 2004, more households in Balochistan and Khyber Pakhtunkwa used government facilities than in Sindh and Punjab.

**Table 3:** Usual source of medical attention reported by households in 2001/2 and 2004

Usual source of medical attention	Weighted percentage (number) of households	
	2001/2 (n=57,075)	2004 (n=53,118)
Nowhere	0.25 (219)	0.06 (42)
Government health facility	29.2 (22,139)	23.9 (18,060)
Private qualified practitioner or facility	45.0 (25,215)	45.1 (21,647)
Private unqualified practitioner	23.8 (8,724)	29.2 (12,476)
NGO or services facility	1.7 (778)	1.8 (893)

**Table 4:** Proportion of households usually using government health facilities for medical attention in 2001/2 and 2004, by province

Province	Weighted percentage (fraction) of households using government health facilities	
	2001/2	2004
Sindh	22.9 (2,696/10,694)	19.7 (2,017/9,902)
Balochistan	50.5 (6,209/12,211)	45.6 (5,837/12,428)
Khyber Pakhtunkhwa	58.6 (7,657/12,850)	54.0 (6,636/11,766)
Punjab	23.8 (5,348/20,832)	17.7 (3,570/19,022)

Taking other variables into account, the proportion of households usually using government health facilities did not change significantly between 2002 and 2004, except in Punjab, where there was a significant *decrease* in the proportion of households usually using government health facilities (ORa 0.66, 99% CIca 0.55-0.80).

In 2004, households in all provinces who said they were satisfied with the government health facilities in their area were more likely to say they usually went to a government facility for medical attention (Table 5). Similarly, in all provinces, those who said they knew how to complain about the usual service they used were more likely to use a government facility for medical attention. However, there were also differences between provinces. In Sindh and Khyber Pakhtunkhwa, vulnerable households were more likely to seek medical attention from government facilities. In Sindh, respondents with formal education were less likely to choose a government facility for medical attention, as were those from households with an educated head in Balochistan. In Punjab, urban households were less likely to use government health facilities.

**Table 5:** Final models from multivariate analysis of variables associated with households usually using government health facilities in 2004, in the four provinces

	ORa	99% CIca
**Explanatory variable/Province**		

**Sindh**		
Respondent with any formal education	0.62	0.48 – 0.80
Vulnerable housholds^1^	1.90	1.42 – 2.84
Know how to complain about usual service	1.47	1.32 – 2.59
Satisfied with government health services	3.22	1.19 – 6.92
**Balochistan**		
Household head with any formal education	0.75	0.59 – 0.97
Know how to complain about usual service	1.49	1.12 – 1.98
Satisfied with government health services	1.85	1.29 – 2.66
**Khyber Pakhtunkhwa**		
Vulnerable households^1^	1.25	1.03 – 1.51
Know how to complain about usual service	1.48	1.21 – 1.81
Satisfied with government health services	1.36	1.05 – 1.76
**Punjab**		
Urban households	0.56	0.30– 0.79
Know how to complain about usual service	1.31	1.11 – 1.68
Satisfied with government health services	2.44	1.90 – 3.61

ORa= adjusted Odds Ratio;

CIca= cluster adjusted confidence interval around the ORa

^1^ Vulnerable households had at least two of three vulnerability factors: poor roof construction, overcrowding, and poor occupation of the main breadwinner.

The variables included in all initial models were: urban/rural location of household, sex of the respondent, education of the respondent, education of the household head, vulnerability status of the household, knowledge of how to complain about usual health services, whether there was a government health facility within 5 Km, satisfaction with government health services in the area.

Focus groups in 2004 offered a number of explanations for why people avoided using government health facilities and recommendations for how to encourage people to use government services. In the following paragraphs, we include quotes in order to illustrate the common points raised by groups.

Nearly all groups across the country cited problems with availability and quality of medicines in government facilities as an important reason for not using these services. They complained that such medicines as were available were fake, expired, or very basic (such as aspirin). They suggested more people would use the government services if they had free medicines available.

“Why should we waste our time when we don’t get any medicine? We go to government facilities to get medicine. We don’t go there to look at the doctor.” *Male Focus Group, Balochistan*

“People will go there if they start getting free medicines.” *Female Focus Group, Sindh*

Many groups also said people avoided government health facilities because of the bad attitude of doctors and other health workers in these facilities; they complained patients were not treated with any respect unless they were rich or influential, and they felt humiliated at the hands of the health workers. Many felt that changing the behaviour of government health workers could be a way to encourage more people to use government health services.

“The doctors treat us like dirt; as if we are worse than animals. But they bend over backwards when a rich person goes to see them. These doctors only know how to lick the feet of the rich.” *Female Focus Group, Balochistan*

“If they stop treating us like animals, and improve their behaviour, we will use their services.” *Female Focus Group, Khyber Pakhtunkhwa*

There was a widespread perception that people avoided government facilities because they believed the quality of medical treatment was sub-standard: doctors not examining patients and prescribing the same medicine for every ailment; the patient's condition worsening after visiting the facility. Lack of staff, equipment and infrastructure also led people to avoid government health facilities.

“Doctors give us paracetamol for every ailment. It seems that there is only one treatment for every problem.” *Male Focus Group, Sindh*

“We do not have doctors and nurses in government hospitals. Our women die during childbirth. Why should we go there?” *Male Focus Group, Sindh*

Some groups mentioned corruption as a reason for people avoiding government facilities. They complained that government medicines are diverted and sold in the market, and doctors and other health workers expect unofficial payments in exchange for providing care. They urged that corruption in government health facilities must be tackled, and some said this could be done by improving management and monitoring of the facilities.

“When we take our children to government facilities, we get expired medicines. The new ones, you see, are sold off in the market.” *Female Focus Groups, Punjab*

“Nurses expect to be paid Rs 25-30 for taking our blood pressure.” *Female Focus Group, Balochistan*

“Doctors should work their correct hours. And, if they don’t, appropriate action must be taken against them.” *Male Focus Group, Sindh*

### Satisfaction of users of government health services

In 2002, considering all provinces together, among those household members who had used government health services in the last three months, nearly two thirds reported they were satisfied with the service they received (Table 6). The proportion satisfied was higher among household members who used other health care providers; the highest satisfaction was among users of private qualified practitioners (Table 6). There was a modest increase in satisfaction among users of government services between 2001/2 and 2004.

**Table 6:** Proportion of service users in last three months who were satisfied with the service they received in 2001/2 and 2004, by type of service used

Type of service used	Weighted percentage (fraction) of users satisfied	
	2001/2	2004
Government health facility	62.9 (10,778/18,707)	66.7 (9,753/15,784)
Private qualified practitioner or facility	84.3 (17,691/21,875)	86.8 (16,280/19,044)
Private unqualified practitioner	76.0 (6,024/7,954)	77.7 (8,940/11,583)
NGO or services facility	77.2 (538/660)	81.0 (597/724)

Satisfaction of government service users varied somewhat between provinces, being higher in Sindh and Punjab than in Balochistan. In all provinces, except Khyber Pakhtunkhwa, there was an increase in satisfaction between 2001/2 and 2004 (Table 7). Taking other variables into account in a multivariate analysis, government services users were significantly more likely to be satisfied in 2004 than in 2001/2 in Sindh (ORa 1.52, 99% CIca 1.03-2.23) and in Punjab (ORa 1.32, 99% CIca 1.10-1.59).

**Table 7:** Proportion of users of government health services in the last three months who were satisfied with the service received in 2001/2 and 2004, by province

Province	Weighted percentage (fraction) of users satisfied with service	
	2001/2	2004
Sindh	69.8 (1,599/2,357)	75.5 (1,330/1,746)
Balochistan	48.0 (2,565/5,427)	55.2 (2,870/5,214)
Khyber Pakhtunkhwa	60.0 (3,725/6,324)	60.0 (3,394/5,688)
Punjab	64.0 (2,767/4,424)	69.3 (2,159/3,136)

In 2004, only 28.3% (3,050/15,685) of service users reported they got all the necessary medicines from the government facility; this varied considerably from only 8.5% in Khyber Pakhtunkhwa to 45.5% in Punjab. Most (82.5%, 13,144/15,480) reported the health worker explained their condition, with little variation between provinces. Users of government health services in 2004 in all provinces were more likely to be satisfied if all the medicines they needed were available from the health facility, and if the health worker gave them an explanation about their illness or condition (Table 8). In Balochistan users were less likely to be satisfied if they were from a vulnerable household, and in Khyber Pakhtunkhwa those from households with an educated head were more likely to be satisfied.

**Table 8:** Final models from multivariate analysis of variables associated with service users being satisfied with the service in 2004, in the four provinces

	ORa	99% CIca
**Explanatory variable/Province**		

**Sindh**		
All medicines available from the facility	3.08	1.18-5.24
Health worker explained about the condition	4.03	2.13-7.62
**Balochistan**		
From vulnerable household^1^	0.76	0.59-0.97
All medicines available from the facility	3.74	2.26-6.20
Health worker explained about the condition	3.27	2.24-4.78
**Khyber Pakhtunkhwa**		
Household head has some formal education	1.29	1.11-1.50
All medicines available from the facility	2.18	1.58-3.02
Health worker explained about the condition	2.67	1.94-3.68
**Punjab**		
All medicines available from the facility	1.79	1.40-2.29
Health worker explained about the condition	2.50	1.69-3.69

ORa= adjusted Odds Ratio;

CIca= cluster adjusted confidence interval around the ORa

^1^ Vulnerable households had at least two of three vulnerability factors: poor roof construction, overcrowding, and poor occupation of the main breadwinner.

The variables included in all initial models were: urban/rural location of household, education of the household head, vulnerability status of the household, availability of medicines in the facility, explanation of the condition, payment for medicines in the facility, and payment to the health worker(s).

### Information from district *nazims* and DCOs

In 2004/5, the field teams interviewed 84 district (elected) *nazims* and 86 (appointed) DCOs or their deputies. Both groups were mostly positive about the concept of devolution. Many *nazims* cited support from their local electorate as important in helping them to provide services in their districts. However, both *nazims* and DCOs described serious difficulties in the practical implementation of devolved government in their districts. The problem was that they had been given the responsibility for providing services in place of the provincial governments, but the funding to do so did not necessarily follow as it should have. Many districts cited long delays in receiving funding, which led to problems with providing basic services, or disagreements about the total amount of funding they were to receive to provide services. Many *nazims* and some DCOs also complained of other hindrance from their provincial government, including, in particular, interference in staff posting and transfers. Their complaints related to all aspects of fulfilling their role, and not only to health services. Many *nazims* in particular perceived that provincial governments were against devolution and were therefore trying to ensure that it would not be fully implemented, and were making life difficult for district governments. Referring to the perceived resistance to devolution from the civil service and provincial governments, one *nazim* explained: “We can improve district government performance only if the bureaucracy and provincial government accept and implement the new local government system in its true spirit” [5].

## Discussion

Pakistan is a country of much diversity and there are important inter-provincial differences. This is reflected in some differences we found between provinces. Nonetheless, the overall finding from the social audits – that there was little improvement in patient views and experience of government health services under devolution – held for all four provinces. We found no evidence in any province that devolution increased the use of government health services. Less than a quarter of households usually used government health services for medical attention in 2004, three years into devolution and even less than in 2001/2. In Punjab, home to two-thirds of the population of Pakistan, there was actually a significant decrease in use of government health services over the period of devolution. The low household use of government health services in 2004 reported here (24%) is very similar to the figure of 23% reported from the Pakistan Social and Living Standards Measurement Survey at around the same time [10]. Low use of government health services was reported in Pakistan prior to devolution [11-13]. The Pakistan National Health Survey completed in 1994 reported that 21% of the public consulted government doctors, while 65% consulted private doctors [12]. In poor communities in Pakistan in 2001, only 21% of reported health care visits were to government facilities [13]; a national survey in 2001-02 reported that only 25% of households went first to a government facility for treatment of childhood diarrhoea [14]. The situation in Pakistan is not unusual: many people in developing countries get their health care mainly from the private sector [15,16], including in South Asia [17,18].

The social audits share the limitation of all cross-sectional studies, that one cannot be certain about the direction of associations. We found few improvements over time. It is possible that there may have been more deterioration over time in the absence of devolution, but we have no supporting evidence for this interpretation.

There is continuing debate about whether to improve health care in developing countries by strengthening the public role in the health system or by engaging with the private sector [19,20]. In Pakistan, devolution did not succeed in drawing the public back to government health services in any province. The public had a generally negative view of government health services at the beginning of devolution (less than a quarter were satisfied with the service in their area) and it would be hard to shift such entrenched attitudes without both big changes in service delivery and an effective communication campaign about this. Among the minority of households who used government services, there was a modest increase in satisfaction with the service received over the three years of devolution. Clearly this was not enough to shift public opinion in favour of using government services.

Teasing out household choice of health care provider, we found that households who were satisfied with the government health services in their area were more likely to use these services. This may reflect local variation in quality of government health services: in places where the service is relatively good, people get to know this and choose to use the service. It suggests that in order to increase use of government health services it is necessary to improve the reputation of the service among both service-users and, critically, among previous non-users. This did not happen during the first three years of devolution. Other factors related to use of government services were education and vulnerability of the households: households with an educated head were less likely to use government services while those in the vulnerable (poorer) category were more likely to use them. A previous study in two districts of Sindh province also found that more educated people were more likely to choose private health care [21]. A study in poor communities of Pakistan found that the poorest people were more likely to use either government facilities or unqualified private practitioners [13]. Our finding that urban dwellers in Punjab were less likely to use government health services probably reflects the greater availability of private alternatives in urban sites. We did not find that distance from the nearest government health facility made a difference to whether households used government health services. Similarly, a study in a poor district of Sindh found that distance from a government health facility was not a determinant of choice of provider for childhood illness, but distance from a private facility was [11]. We found that households were more likely to use government health services if they knew how to complain about their usual health service; this might be a positive finding if it reflects that users of government health services receive more information about how to complain than users of other services.

Despite challenges of measurement and concerns over comparability and repeatability, patient satisfaction surveys are recognised as important for measuring service improvements relevant to patients [22]. In both our surveys, users of private services (including the services of unqualified practitioners) were more satisfied than users of government services, consistent with other reports from developing countries [17,18,23,24]. Some authors have argued that the basis for satisfaction is shaky and patients respond mainly to being treated with respect and kindness and are not in a good position to judge actual medical appropriateness of the treatment they receive [25]. Confronted with evidence of low satisfaction of service users, health workers may also claim that patients' satisfaction ratings are flawed and based on factors unrelated to “good medical practice” [26]. We found satisfaction among users of government services was related to two main factors: availability of medicines and whether patients were given an explanation of their condition. While this provides a guide for what might make patients more satisfied, actually improving these aspects of service delivery is complex and requires action at multiple levels. For example, reported lack of medicines could relate to inadequate supply of medicines to the facilities, diversion of the supplied medicines, and patient misconceptions about appropriate treatments. In theory, if devolution gave greater local control and accountability, it should be easier to examine the reasons for lack of medicines at the point of patient contact and correct the problems. In practice, this did not happen in the first few years of devolution. Under devolution, district governments should also have been better able to influence health workers' behaviour, as health workers became employed at district level. However, in practice, district *nazims* complained of considerable interference from higher levels of government in staff transfers and disciplinary issues.

There were problems with the implementation of devolution in practice. Under the Local Government Ordinance, health services became the responsibility of locally elected district governments [2,3]. The district *nazims* and DCOs interviewed as part of the social audit described difficulties they faced in delivering health and other services because of inadequate and delayed funding reaching them from the provincial governments. A 2007 report concluded that devolution had not achieved improvements in health indicators and pointed to continuing problems with implementation of devolution [27]. The report noted that provincial governments had transferred responsibility for delivery of major public services to district governments but without also transferring the necessary funding. A World Health Organisation 2007 health system review mission noted a mixed picture for health services under devolution, and pointed to a need for improved managerial and planning skills at district and provincial levels and clarification of changed roles and responsibilities under devolution [28]. A 2006 report on the impact of devolution on health care and education pointed to a number of reasons why devolution had not improved health care, including lack of adequate administrative and fiscal decentralisation, lack of district government capacities to deliver services, and continuing procurement problems [29]. An earlier study by a group of donors found some evidence of improvements in supply of medicines, but documented a number of problems with the implementation of devolution in practice [30].

Devolved local governments should be in a better position to consult the public within districts and to plan and deliver health and other services based on good local evidence. However, their ability actually to improve services, based on the findings from such consultations depends, not least, on having adequate human and financial resources. In the first three years of devolution in Pakistan, we supported several district governments to use social audit methods to seek evidence about priority health issues across their districts and to plan improved health and other services based on the evidence [31-35]. This process involved extensive capacity building for local government personnel and was well received in the districts; the district governments designed their own social audits, and identified officers who conducted the surveys and took part in the analysis, with support from CIET. The actual process of the social audit was not expensive, and would be even less so over time. Unfortunately, the funding districts received from their provincial governments to provide services was barely enough to cover their recurrent costs and the districts implementing these local social audits were therefore not able to implement their planned evidence-based improvements to services, even at modest cost.

## Conclusion

During the first three years of devolved local government in Pakistan, government health services did not improve from the point of view of the public, who continued to choose private health services and to rate these services more positively than government services. This is not to say that devolution could not make a difference to health services if fully and properly implemented. An ongoing social audit process both nationally and locally could provide a basis for accountability under devolution, taking monitoring of services beyond internal review and focusing on the impact of services on the intended beneficiaries.

## List of abbreviations used

CIca: Cluster adjusted confidence interval; DCO: District Coordinating Officer; ORa: Adjusted Odds Ratio

## Competing interests

The authors declare they have no competing interests.

## Authors' contributions

UA assisted with the data collection and analysis of the surveys, conducted the analysis for this paper, and drafted the paper. AC designed and supported the surveys and their analysis and reporting, contributed to the analysis for this paper and helped to draft the paper. KO assisted with design, led implementation of the surveys, contributed to their analysis and reporting, and assisted with the drafting of the paper. NMA, AK, and UUC assisted with design and implementation of the surveys, contributed to analysis and reporting, and assisted with the drafting of the paper. NA designed the surveys, supported their analysis and reporting, and supported the drafting of the paper.
